# PM_2.5_ Exposure Suppresses Dendritic Maturation in Subgranular Zone in Aged Rats

**DOI:** 10.1007/s12640-017-9710-4

**Published:** 2017-03-08

**Authors:** Lewis Cheng, Way K.W. Lau, Timothy K. H. Fung, Benson W.M. Lau, Bolton K.H. Chau, Yutong Liang, Zhe Wang, Kwok Fai So, Tao Wang, Chetwyn C. H. Chan, Tatia M. C. Lee

**Affiliations:** 1 The State Key Laboratory of Brain and Cognitive Sciences, The University of Hong Kong, Pokfulam, Hong Kong; 2 Laboratory of Cognitive Affective Neuroscience, The University of Hong Kong, Pokfulam, Hong Kong; 3 Laboratory of Neuropsychology, The University of Hong Kong, Pokfulam, Hong Kong; 4 Applied Cognitive Neuroscience Laboratory, Department of Rehabilitation Sciences, The Hong Kong Polytechnic University, Hung Hom, Kowloon, Hong Kong; 5 Department of Civil and Environmental Engineering, The Hong Kong Polytechnic University, Hung Hom, Kowloon Hong Kong; 6 Guangdong-Hong Kong-Macau Institute of CNS Regeneration, Jinan University, Guangzhou, China; 7 Department of Ophthalmology, Li Ka Shing Faculty of Medicine, The University of Hong Kong, Pokfulam, Hong Kong; 8 Institute of Clinical Neuropsychology, The University of Hong Kong, Pokfulam, Hong Kong

**Keywords:** Fine particulate matter, Neurogenesis, Ammonium sulfate, Dendritic complexity, Air pollution

## Abstract

Detrimental effects of long-term inhalation of fine particulate matter (PM_2.5_) on the pulmonary and cardiovascular systems have been widely reported. Recent studies have shown that exposure to PM_2.5_ also causes adverse neurocognitive effects. This study investigates the effects of inhaled ammonium sulfate, which is a major compound of inorganic air pollutants in PM_2.5_, on adult neurogenesis in aged Sprague-Dawley rats. A total of 20 rats were randomly assigned to experimental (*n* = 10) and control (*n* = 10) conditions, wherein they were exposed to either ammonium sulfate or sham air for 2 h per day and for 28 consecutive days. It was observed that ammonium sulfate inhibited the maturation process and diminished dendritic complexity of immature neurons in the subgranular zone (SGZ) of the hippocampus significantly, although the number of neural stem cells or the rates of differentiation were comparable between the two groups. Our findings provide clear evidence on the direct relationship between air quality and advantageous neurogenesis. Exposure to PM leads to specific adverse effects on the maturation process during neurogenesis.

## Introduction

Fine particulate matter (PM) has raised public concern regarding its impacts on our health. Long-term inhalation of PM incurs harmful effects not only on the pulmonary and cardiovascular systems (Brook et al. 2010; Mills et al. 2009) but also in the central nervous system. According to the U.S. Environmental Protection Agency (EPA), PM is defined as a mixture of extremely small particles and liquid droplets comprising of a number of components, including “acids (such as nitrates and sulfates), organic chemicals, metals, soil or dust particles, and allergens (such as fragments of pollen and mold spores)” (The U.S. Environmental Protection Agency 2003). Exposure to PM has been shown to diminish neurogenesis. For instance, reduced neurogenesis in the subgranular zone (SGZ) of the hippocampus has been observed in mice that were treated with PM from a diesel exhaust for 6 h, when compared with those subjected to filtered air control (Costa et al. 2015a). The adverse effects of PM exposure on neurogenesis would inevitably affect cognitive and affective functionings. According to literature, significant decline in working memory, spatial orientation, and verbal learning were shown in human subjects residing in environments polluted with fine particles less than 2.5 μm in diameter (PM_2.5_) (Ailshire and Crimmins 2014; Gatto et al. 2014; Tonne et al. 2014). Following this school of thought, the elderly population would be particularly vulnerable to the PM_2.5_ effects, given the fact that their brain functioning is already under the threat of age-related neurodegenerative changes. This study verifies the direct relationship between PM_2.5_ exposure and the process of neurogenesis in the aging of the brain.

Adult neurogenesis refers to the different stages of neuronal development during adulthood, which include the proliferation of neural stem cells, differentiation of neuroblasts, and subsequent maturation of immature neurons (Huang and Reichardt 2001; Zhao et al. 2008). The subventricular zone (SVZ) near the lateral ventricle and the SGZ in the dentate gyrus of the hippocampus are the two specific neurogenic brain regions that possess neural stem cells in adults that are essential for neurogenesis under normal conditions (Gould et al. 1999; Pencea et al. 2001; Snyder et al. 2012; Spalding et al. 2013). Neurogenesis within these two traditional neurogenic sites has garnered great attention in the neuroscience field, since new cells produced from these sites have been shown to integrate into different networks of the brain functionally. This plays important roles in maintaining sensory and cognitive functions across the adulthood [reviewed by (Lledo et al. 2006)]. For example, neurons arisen from the SGZ can be incorporated into the existing neural network of granule cells in the dentate gyrus of hippocampus (Ge et al. 2006; van Praag et al. 2002), which contributes to hippocampus-dependent functions, such as learning and memory (Kee et al. 2007). In a normal process of aging, significant reduction in cell proliferation in both adult SGZ and SVZ can be observed [reviewed by (Rossi et al. 2008)], which takes part in the decline in both sensory and cognitive functions during normal aging (Drapeau et al. 2003; Kuhn et al. 1996; Molofsky et al. 2006).

Ammonium and sulfate are the two dominant species of inorganic air pollutants in PM_2.5_ in Hong Kong (Huang et al. 2014) and in mainland China (Zhang et al. 2015). They may impair neurogenesis. Ammonia (1–10 mM) has been shown to induce free radical production (Murthy et al. 2001) and astrocyte swelling (Jayakumar et al. 2009; Panickar et al. 2009) in primary cultures of rat astrocytes. This is highly relevant because astrocyte and oxidative stress are the key modulators of adult neurogenesis (Haussinger and Gorg 2010). These findings suggest that ammonium sulfate may be a potential active component that can modulate neurogenesis in the SGZ and SVZ of the brain. This study investigates the effects of inhaled ammonium sulfate in the form of PM_2.5_ on adult neurogenesis in aged rats. We hypothesize that inhalation of ammonium sulfate would produce a general inhibitory effect on neurogenesis, i.e., on neural stem cell proliferation, neuroblast differentiation, and neuron maturation in the neurogenic sites.

## Materials and Methods

### Animals

Sprague-Dawley (SD) rats used in this study were retired breeders purchased from the Centralised Animal Facilities (CAF) at The Hong Kong Polytechnic University. They were singly housed in standard-size cages provided by the CAF with woodshaving bedding under 12 h light/12 h dark cycles. Standard irradiated labdiet and water were provided ad libitum by the CAF. Twenty aged male SD rats (9 to 13 months old) were randomly assigned either to an experimental PM_2.5_ group (*n* = 10) or to a sham air control group (*n* = 10). Only male rats were studied, because we would like to avoid potential influence from sex hormones such as estrogen. The age of the two groups did not differ significantly [(mean ± SEM: 10.30 ± 0.496 vs 10.10 ± 0.433 months, control vs PM_2.5_), *U* = 52.5; *p* = 0.87]. All experimental procedures had been approved by Animal Subjects Ethics Sub-Committee of The Hong Kong Polytechnic University.

### Treatment

**Fig. 1:**
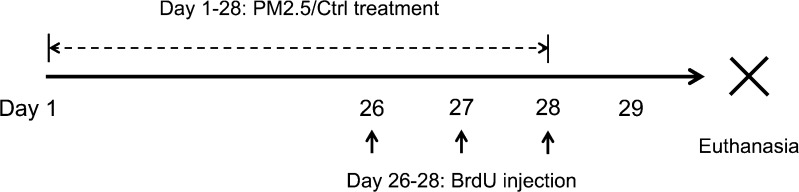
Treatment schedule of the study. Experimental (*PM*_*2.5*_) or control (*Ctrl*) treatment lasted for 4 weeks, and euthanasia was conducted on day 29. Bromodeoxyuridine (*BrdU*) was injected on the last 3 days of treatment to label proliferating cells

The animals were exposed to either ammonium sulfate (PM_2.5_ group) or sham air (control group) in a group of five inside a plastic chamber (size: W38 cm × L54 cm × H20 cm) with woodshaving bedding for 2 h per day in the morning, 7 days per week for 28 days. The bedding was changed after each treatment, and the apparatus was cleaned between uses. The PM_2.5_ of ammonium sulfate was produced in a collision nebulizer (NSF Model–CN 311, from BGI, Inc.) by dissolving ammonium sulfate in distilled water (110 g/L), and then applying approximately 30 psi pressure, and with dehumidified air as carrier gas. The air coming from the nebulizer was then mixed with dry air to maintain the humidity at 30%, and this is to ensure that the humidity was below the deliquescence point of ammonium sulfate. The mixed gas was then passed on to a cyclone, where only particles with diameter less than 2.5 μm remained in the air. The aerosol outlet was then connected directly to the inflow of the treatment chamber, where an outflow was available in another end of the chamber so that the pressure, concentration of oxygen, and carbon dioxides inside the chamber would be similar to that of the ambient condition. The average concentration of aerosol was 595 μg/m^3^. For the control group, the treatment conditions were the same, except that no ammonium sulfate was added. Bromodeoxyuridine (BrdU, Sigma, St. Louis, MO, USA) was injected intraperitoneally (50 mg/kg) to each rat on day 26, 27, and 28. The animals were euthanized by intraperitoneal injection of overdose sodium pentobarbital (100 mg/kg) on day 29 (Fig. 1).

### Histology

#### Preparation of Brain Sections

Animals were perfused with normal saline prior to 4% paraformaldehyde in a 0.1 M phosphate buffer transcardially. After the perfusion, the brains were dissected and post-fixed in 4% paraformaldehyde at 4 °C overnight. Cryosections of the right hemisphere that were of 40 μm thick were prepared in 1-in-12 series using a cryostat. Coronal brain sections, including the SVZ (for the proliferation assay) and the SGZ of the hippocampus (for the proliferation, differentiation, and maturation assays), were affixed on glass slides coated with gelatin at room temperature before immunostaining.

#### Immunoperoxidase Staining

Neural stem cells were determined by BrdU immunoperoxidase staining. Antigen retrieval was carried out by incubating the brain sections in citric acid (pH 6.0) at 90 °C for 25 min, followed by incubating them in 2 N hydrochloric acid (HCl) at 37 °C for 30 min, and then in borax acid buffer (pH 8.5) at room temperature for 15 min. The brain sections were then incubated in mouse anti-BrdU primary antibody (1:1000, Roche) at room temperature overnight, followed by biotinated goat anti-mouse secondary antibody (1:200, Dako) for two more hours. Immature neurons were determined by doublecortin (DCX) immunoperoxidase staining. The procedures were similar to that of BrdU staining, except that antigen retrieval was done without the incubation in HCl and borax acid buffer. Rabbit anti-DCX primary antibody (1:300, CST) and biotinated goat anti-rabbit secondary antibody (1:200, Dako) were used in DCX immunoperoxidase staining. After secondary antibody incubation, all slides were then incubated in Avidin-Biotin complex solution for 2 h followed by freshly prepared diaminobenzidine (DAB, Sigma-Aldrich). Proliferative neural stem cells labeled by BrdU antibody and immature neurons labeled by DCX antibody were counted using Nikon series Eclipse H600L microscope.

#### Immunofluorescence Staining

Neuroblasts were determined by BrdU and DCX double labeling. The brain sections were incubated in mouse anti-BrdU primary antibody (1:1000, Roche) and rabbit anti-DCX primary antibody (1:300, CST) at room temperature overnight, and this was followed by Alexa Fluor 488-conjugated goat anti-mouse and Alexa Fluor 568 goat anti-rabbit antibodies (1:400, Molecular Probe) for two more hours. Nuclei were counterstained by DAPI. The fluorescence signals were captured using Nikon series Eclipse H600L microscope.

#### Cell Counting

Quantification of cells was performed by an experimenter, who was blind to the treatment condition. For proliferative neural stem cells and immature neurons count, unbiased stereology with the fractionator method (Sanchez-Vidana et al. 2016) was used to measure the number of neural stem cells in the SVZ, and neural stem cells and immature neurons in the SGZ of the hippocampus. Cells were counted for every 12th section of the brains with the Stereo Investigator system (version 11, MBF Bioscience) by a camera interfaced with Nikon series Eclipse H600L microscope coupled with a motorized stage. Only DCX-positive cells with tertiary dendrites were counted as immature neurons. For differentiating neuroblasts, neuronal differentiation was indicated by the percentage of BrdU-positive cells expressing DCX (Sanchez-Vidana et al. 2016).

### Sholl Analysis

Seven to 13 DCX-positive cells in with tertiary dendritic branches of each animal were selected randomly, microphotographed, and traced at ×400 magnification. The photographs were imported into ImageJ software (NIH) for Sholl analysis. Concentric circles were drawn from the cell body of DCX-positive cells with 10 μm apart, and the number of interceptions between the dendrites of the cells and the concentric circles were recorded. The number of interceptions obtained from 7 to 13 cells was averaged to generate a data point per distance from soma for each animal. The mean values from 10 animals per group were used to perform statistical analysis and plot graph. A higher number of interceptions indicates a higher dendritic complexity.

### Statistical Analyses

Normality was tested by the Shapiro-Wilk test. Mean differences between PM_2.5_ and control groups were compared by using the two-tailed independent Student’s *t* test or the Mann-Whitney *U* test, where appropriate. Changes in body weight were determined by linear regression controlled for the body weight at baseline. A statistically significant difference was indicated when *p* < 0.05.

## Results

### Body Weight

**Fig. 2:**
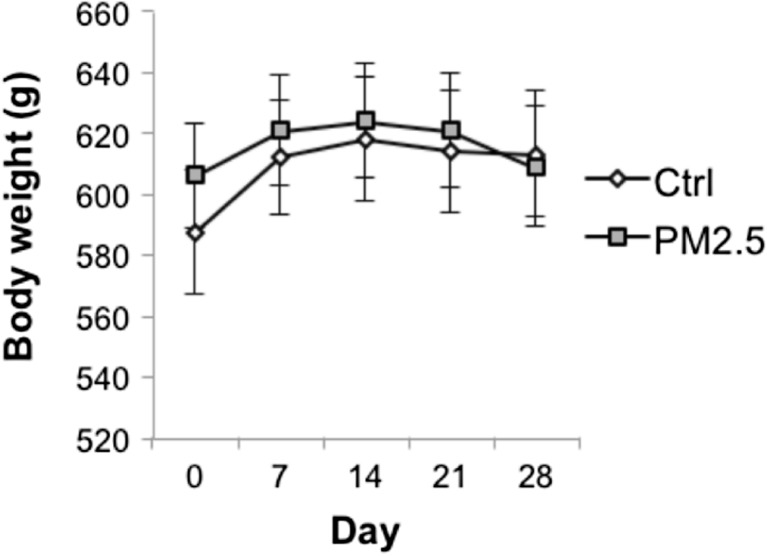
Variations in body weight of the PM_2.5_ and control groups throughout treatment. Data are presented as mean ± SEM

There was no significant group difference in the body weight [(mean ± SEM: 587.00 ± 20.24 vs 605.80 ± 16.90 g, control vs PM_2.5_), *t*(18) = −0.147; *p* = 0.885] at baseline. The body weight of both PM_2.5_ and control groups increased from day 0 to 14, but decreased after day 14 for the PM_2.5_ group. The body weight remained the same after day 14 for the control group (Fig. 2). The average body weight gain calculated by subtracting the body weight from day 28 to day 0 (in grams) of the PM_2.5_ group (mean ± SEM: 2.70 ± 6.64 g) was significantly less than that of the control group [(mean ± SEM: 25.70 ± 3.28 g), *t*(17) = −3.127; *p* = 0.006, controlled for the effect of the body weight at baseline].

### Histology

#### Maturation and Dendritic Growth

**Fig. 3:**
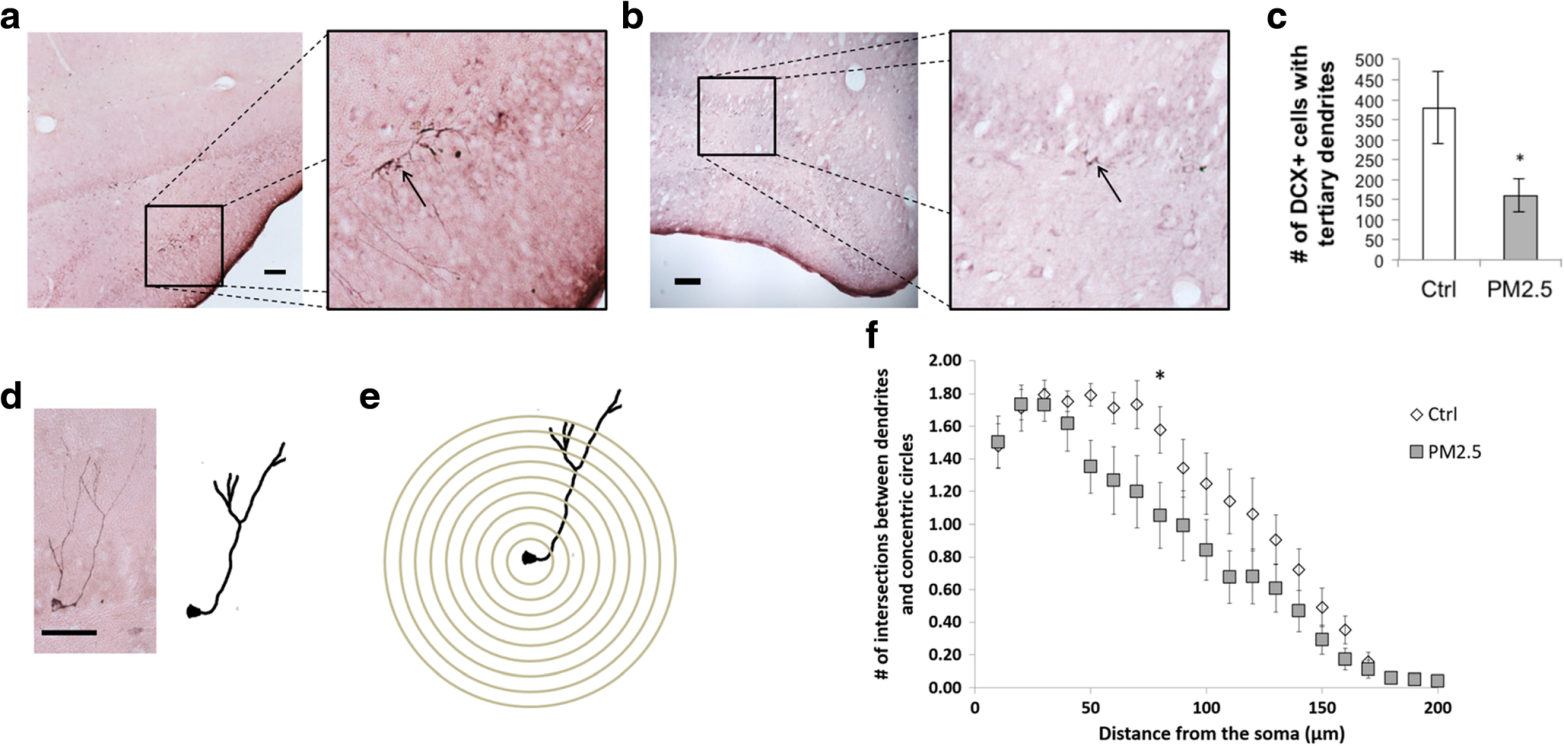
PM_2.5_ treatment impaired maturation and dendritic growth in the SGZ of the hippocampus. Photomicrographs of the SGZ showing doublecortin (DCX)-positive cells in **a** control and **b** PM_2.5_ groups. Magnification = ×100. *Scale bar* = 100 μm. **c** The number of DCX-positive cells with tertiary dendrites was significantly lower in the PM_2.5_ group, compared to the control. **d** A sample microphotograph of a DCX-positive cell (*left*), and its traced outline for the Sholl analysis (*right*). Magnification = ×400. *Scale bar* = 50 μm. **e** Graphical representation of the Sholl analysis: *concentric circles* were drawn from the cell body of DCX-positive cells with 10 μm apart. **f** Sholl analysis of DCX-positive cells with tertiary dendrites showed that the PM_2.5_ group had significantly less branched points than the control group at 80 μm from the soma. Data are presented as mean ± SEM. *p < 0.05

The maturation of the newborn neurons was assessed by quantification of DCX-positive neurons (Fig. 3). The PM_2.5_ group (mean ± SEM: 160.60 ± 39.25) had significantly fewer DCX-positive neurons with tertiary dendrites than that of the control group [(mean ± SEM: 378.50 ± 89.30), *U* = 81; *p* = 0.019; Fig. 3a–c]. This suggests the decrease in maturation of newborn neurons after a 28-day exposure to ammonium sulfate. Sholl analysis was performed for a more fine-grained investigation of the dendritic growth of those new neurons (Fig. 3d **)**. The results showed that the PM_2.5_ group, when compared with the control group, had a fewer number of intersections from 50 to 160 μm. Significant fewer number of intersections were observed at 80 μm [(mean ± SEM: 1.58 ± 0.143 vs 1.05 ± 0.202, control vs PM_2.5_), *U* = 77; *p* = 0.034] from the soma of DCX-positive neurons with tertiary dendrites in the PM_2.5_ group, compared to that of the control group. Therefore, it can be suggested that the dendritic complexity of immature neurons in the hippocampal SGZ decreased after exposure to ammonium sulfate for 28 days.

#### Differentiation

**Fig. 4:**
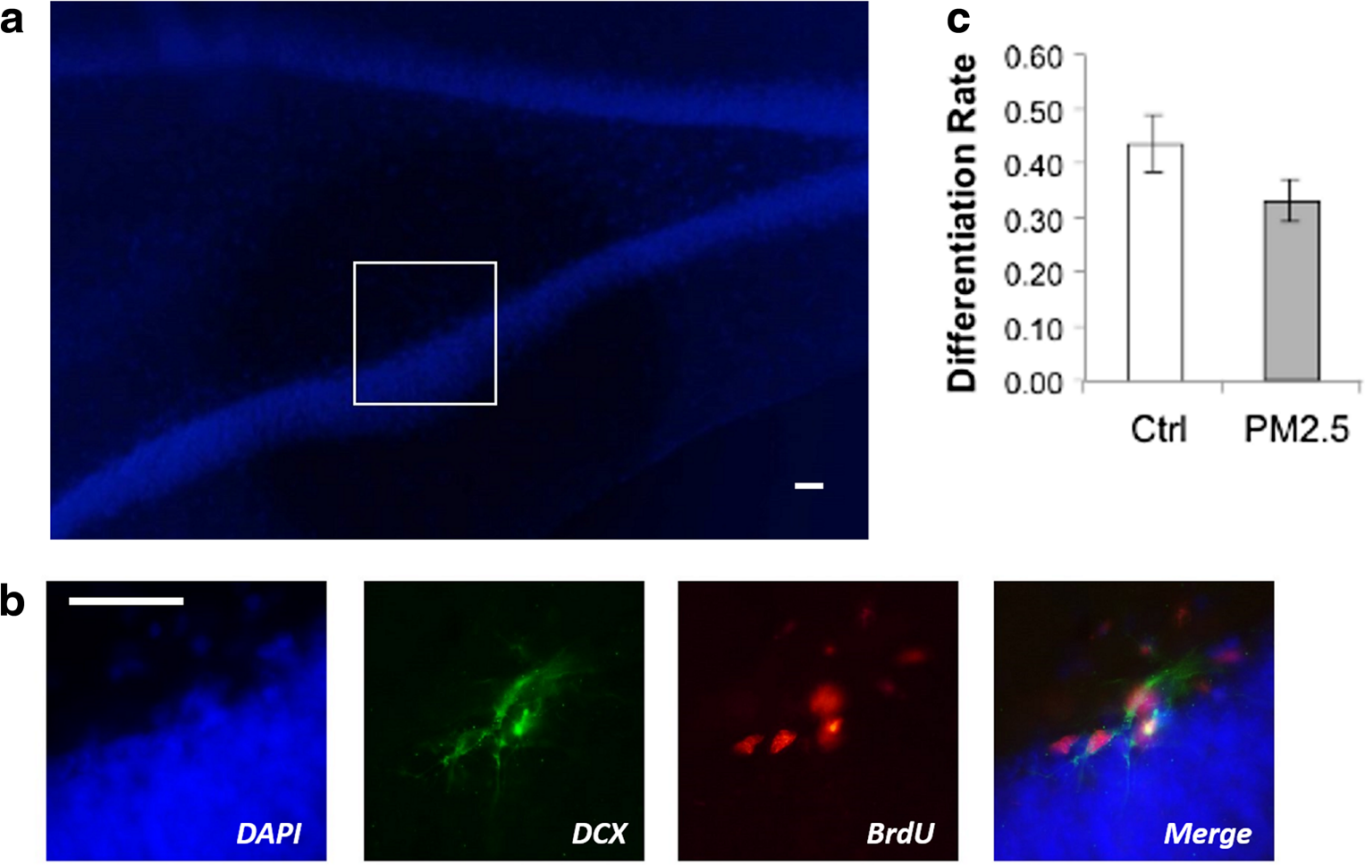
Differentiation of neuroblasts in the subgranular zone (SGZ) of the hippocampus was not affected by PM_2.5_ treatment in aged rats. **a** DAPI staining of the SGZ. Magnification = ×100. *Scale bar* = 100 μm. **b** Immunofluorescent photomicrographs of the SGZ showing co-labeling of cells with bromodeoxyuridine (BrdU, *red*) and doublecortin (DCX, *green*). Magnification = ×400. *Scale bar* = 50 μm. **c** PM_2.5_ treatment did not significantly affect SGZ neuroblast differentiation. Data are presented as mean ± SEM

The proportion of BrdU-positive neurons that expressed DCX in the SGZ was examined to identify new neural stem cells that differentiated into neuroblasts (Fig. 4). There were no significant differences between the PM_2.5_ (mean ± SEM: 0.331 ± 0.0398) and the control groups [(mean ± SEM: 0.439 ± 0.0513), *t*(18) = −1.668; *p* = 0.113; Fig. 4a–d]. This suggests that the differentiation rates of the new neural stem cells were comparable between the two groups.

#### Proliferation

**Fig. 5:**
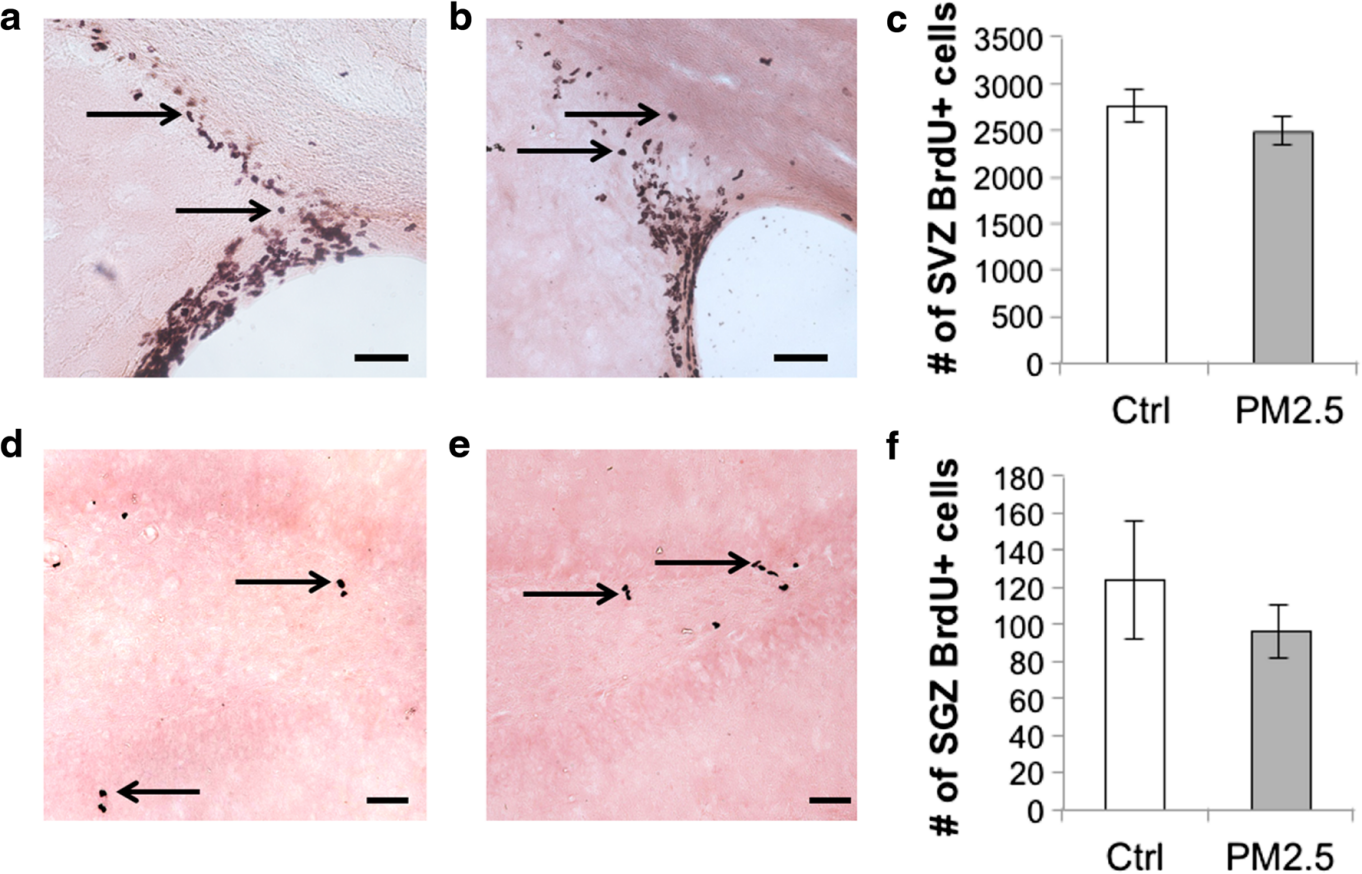
Proliferation of neural stem cells in the subventricular zone (SVZ) of the lateral ventricle and the subgranular zone (SGZ) of the hippocampus was not affected by ammonium sulfate in aged rats. Photomicrographs of the SVZ showing bromodeoxyuridine (BrdU)-positive cells in **a** control and **b** PM_2.5_ groups. **c** There was no significant group difference in neural stem cell proliferation in the SVZ. Photomicrographs of SGZ showing BrdU-positive cells in **d** control and **e** PM_2.5_ groups. **f** There was no significant group difference in neural stem cell proliferation in the SGZ. *Scale bar* = 50 μm. Data are presented as mean ± SEM

To test the effects of PM_2.5_ of ammonium sulfate exposure on the proliferation of neural stem cells, BrdU-positive cells were counted in the SVZ and the SGZ of the hippocampus (Fig. 5). There were non-significant differences between the control and the PM_2.5_ groups in the number of BrdU-positive cells in either the SVZ [(mean ± SEM: 2754.92 ± 178.09 vs 2491.20 ± 147.78, control vs PM_2.5_), *t*(18) = −1.832; *p* = 0.084; Fig. 5a–c] or the SGZ [(mean ± SEM: 123.80 ± 31.31 vs 96.30 ± 13.90, control vs PM_2.5_), *U* = 51; *p* = 0.971; Fig. 5d–f]. This suggests that exposure to ammonium sulfate for 28 days did not inhibit neural stem cells proliferation.

## Discussion

The key findings of this study were that there was a significant reduction in the maturation of newborn neurons and their dendritic complexity after a 28-day exposure to PM_2.5_ of ammonium sulfate. The non-significant group differences in the neural stem cells proliferation or neuroblast differentiation in the neurogenic sites suggested that exposure to PM_2.5_ produces specific effects on the different stages of neurogenesis, rather than general effects. Also, stem cells proliferation and neuroblast differentiation were better preserved than neuron maturation (Amrein et al. 2011) when the aged rats were exposed to ammonium sulfate.

Maturation of hippocampal neurons is essential to the formation of functional synapses. Hence, it is vital to the maintaining of proper hippocampus-dependent functions, such as learning and memory and mood regulation (Costa et al. 2015b). Reduction in hippocampal neurogenesis, including inhibition of the maturation process of neurons (Zeng et al. 2016), has been regarded as one of the mechanisms underpinning aging-related major cognitive impairments, such as the Alzheimer’s disease [AD, (Lazarov et al. 2010)]. In addition, exposure to PM_2.5_ has been shown to accelerate cognitive declines in humans (Ailshire and Crimmins 2014; Gatto et al. 2014; Tonne et al. 2014). Findings from a preclinical study demonstrated a reduction in the dendritic branching of hippocampal neurons in CA1 and CA3 regions after prolonged exposure to a mixture of ambient PM_2.5_ from the environment for 10 months (Fonken et al. 2011). Our findings further support the view that ammonium sulfate can be one of the major components in PM_2.5_ that would impair dendritic complexity. Moreover, chronic exposure to ammonium sulfate in the air may be a risk of major cognitive impairments via inhibition of neuronal maturation.

Unlike a previous report that pointed out acute exposure to PM_2.5_ (6 h) significantly reduced the number of BrdU-positive cells in the SGZ of the hippocampus in adult mice (Costa et al. 2015a), no significant differences in BrdU-positive cell count in either the SVZ or the SGZ between the PM_2.5_ and control groups were observed in this study. This may be attributed to the use of a mixture of ambient PM_2.5_ from diesel exhaust in the aforementioned study (Costa et al. 2015a). The toxic effect from the mixture of PM_2.5_ may be driven by compounds other than ammonium sulfate, but this requires further confirmation. Furthermore, the aforementioned study and this study employed different strains and age of animals. Reduction of cell proliferation was previously reported in adult mice (Costa et al. 2015a) in the aforementioned study, whereas this study looked into aged SD rats. Faster maturation of neurons in rats has been reported, as compared to that in mice within the hippocampus (Perfilieva et al. 2001). This suggests that the underlying mechanisms of neural stem cell proliferation could be different across different strains of the same animal. Furthermore, the effect of PM_2.5_ on adult neurogenesis may be different in adults and in the elderly respectively, given that adult neurogenesis is much reduced in normal aging. Our finding suggests that inhalation of ammonium sulfate exhibits specific inhibitory effect on neuron maturation, rather than a general inhibitory effect on neurogenesis in the SGZ of the aged brain.

There are several limitations in the present study. Firstly, we adopted a design of sub-chronic exposure to a single dosage of ammonium sulfate. Hence, the dose-dependent effects of ammonium sulfate on adult neurogenesis cannot be deduced. Secondly, only male rats were evaluated in the current study. Whether ammonium sulfate exerts a general or a gender-specific effect on neurogenesis requires future studies that evaluate the effect in both genders. Lastly, we reported an inhibitory effect of ammonium sulfate on the maturation of newborn neurons in the SGZ of the hippocampus, which may indicate a decline in hippocampus-dependent functions. However, behavioral data reflecting impaired maturation of neurons after the treatment is currently absent. More studies are warranted to investigate the behavioral outcomes driven by ammonium sulfate-reduced adult neurogenesis in the hippocampus.

In conclusion, our findings support the hypothesis that ammonium sulfate is an active compound in PM_2.5_ that can diminish adult neurogenesis in the SGZ by inhibiting the maturation process of newborn neurons in the aged brain.
